# Effect of straw reinforcement on the shearing and creep behaviours of Quaternary loess

**DOI:** 10.1038/s41598-021-99318-5

**Published:** 2021-10-07

**Authors:** Zhong-Fei Xue, Wen-Chieh Cheng, Lin Wang

**Affiliations:** 1 School of Civil Engineering, Xi’an University of Architecture and Technology, Xi’an, 710055 China; 2 Shaanxi Key Laboratory of Geotechnical and Underground Space Engineering (XAUAT), Xi’an, 710055 China

**Keywords:** Natural hazards, Engineering

## Abstract

In addition to the shearing behavior of soil, the creep character is also considered crucial in determining the long-term shear strength. This especially holds true for the loess that possesses the metastable microstructure and is prone to landslide hazards. This study explored the potential application of straw reinforcement to enhance the shearing and creep properties of the Quaternary loess. The mechanism responsible for the straw reinforcement to elevate the peak shear strength was revealed. Furthermore, three creep characters, namely attenuating creep, non-attenuating creep, and viscous flow were identified in this study. The unreinforced and reinforced specimen behaved in a different manner under identical shear stress ratio condition. The reinforced specimen was superior in limiting the particle relative movement within the shear plane than the unreinforced specimen. The chain reaction of interparticle contact loss, accompanied with excessive viscous displacement, rapid weakening of creep resistance, and eventually accelerated creep displacement, provided an evidence for the formation mechanism of slow-moving landslide. The long-term shear strength using the isochronal stress–strain relationship may be used for optimising the design of high-fill embankment works.

## Introduction

The Chinese Loess Plateau, located in northwest region of China, is the main distribution area of China’s loess and has been prone to landslides^1–9^. The loess tends to behave in an unstable manner when subjected to external disturbances due to its metastable structure, high porosity, and high water sensitivity^10–16^. Therefore, geological disasters like loess landslides have rapidly increased in this region following an increase in engineering activities^17–21^. The shear zone is the weakest layer of a landslide, and its time-dependent creep behavior leads to an inevitable reduction in the strength^22–29^. The benefits of the fiber-reinforced soil are becoming widely accepted as it can control the slope stability and avoid landslide disaster by increasing the shear resistance of soil^30–33^. Fatahi et al.^34,35^ investigated the peak strength and residual strength of soft clay reinforced with fiber and cement by unconfined compressive strength test and found that the addition of fiber increased the residual strength and changed the brittle cement treated clay to a more ductile material. Lian et al.^36^ investigated the shear strength of loess reinforced with root by performing consolidated-undrained triaxial tests, and it was found that the increased internal cohesion contributed the most to the increase in strength of reinforced specimen. Choobbasti et al.^37^ investigated unconfined compressive strength test of soft clay in a construction site reinforced with nano calcium carbonate and carpet waste fibers by performing unconfined compressive strength tests and unconsolidated undrained (UU) triaxial tests. They found that the addition of nanoparticles to the clayey soil reduced its liquid limit and increased its plastic limit whereas the addition of carpet waste fibers to the clayey soil increased the residual strength significantly. The studies above primarily explored the potential for insertion of plant root and barley straw to improve the shear strength of natural soils either using direct shear test or triaxial shear test towards controlling strata erosion. However, studies exploring the shearing behavior versus time for reinforced loess soils when subjected to different magnitudes of loads and mechanism of strength improvement are remarkably limited.

Despite extensive research on the reinforced loess strength, there have been a relatively limited number of studies on the creep properties of reinforced loess so far. For example, Xin et al.^38^ conducted a kinematic analysis of a large landslide on the Loess Plateau in Baoji, China, and it was concluded that the creep movement of the sliding zone is responsible for the landslide. Nowadays, a significant body of research has indicated that the mechanical behavior of landslide is related not only to its peak strength but also to its residual strength and long-term shear strength^39–46^. Wang et al.^40^ studied creep properties of clastic soil in a reactivated slow-moving landslide in the Three Gorges Reservoir Region, China, and it was found that the critical creep stress that triggers the creep failure is slightly lower than the peak shear strength but much larger than the residual strength. Wen et al.^22^ investigated the effect of gravel content on creep behavior of clayey soil at residual state. Contrary to the phenomenon observed by Di et al.^47,48^, Wen et al.^44^ found that the presence of gravel does have a notable effect on creep behavior of clayey soil. Loess and wheat straw which are deemed as the agricultural solid wastes obtained after harvesting of wheat grains are easily accessible in the Chinese Loess Plateau. Loess-straw mixture may be considered as an alternative backfill material to control landslides. However, there is currently limited knowledge on the creep behavior, creep strength characteristics and creep mechanism of loess reinforced with waste straw. The main objectives of this study were: (a) to investigate the shearing behavior of the unreinforced and reinforced specimen under stress-controlled direct shear, (b) to characterise the creep behaviour using the creep displacement, vertical displacement, and creep rate versus elapsed time relationships, and (c) to reveal the formation mechanism of a slow-moving landslide, in order to optimise the design of high-fill embankment works by the long-term shear strength.

## Materials

### Testing materials

**Figure 1:**
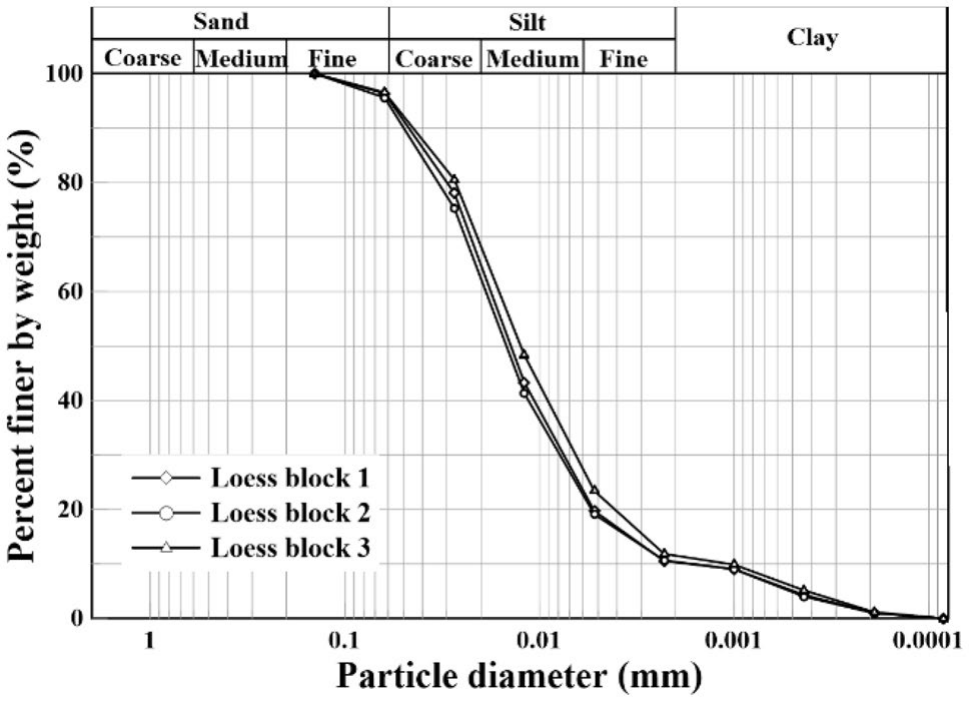
Particle-size distribution curves for the natural loess.

The natural loess blocks in this study were sampled from Lantian county, which is located at a 22 km distance to the southeast of Xi’an city, and transported back to Shaanxi Key Laboratory of Geotechnical and Underground Space Engineering (XAUAT) for preparing a series of large direct shear specimens of 305 mm × 305 mm × 100 mm. The physical parameters of the specimens are summarized in Table 1. Their particle-size distribution curves are shown in Fig. 1. The natural loess was classified as low plasticity clay (CL) according to the Unified Soil Classification System (USCS).

**Table 1:** Physical properties of Quaternary loess.

Sample ID	*C*_u_	*C*_c_	%clay	%silt	%sand	γ_d_ (kN/m^3^)	PL (%)	LL (%)	G_s_	USCS symbol
S1a	9.26	1.94	10.0	83.4	6.6	13.72	18.9	32.0	2.69	CL
S1b	10.48	2.13	11.0	81.1	7.9	13.60	19.4	30.3	2.68	CL
S1c	15.26	2.79	10.0	84.0	6.0	13.83	18.8	31.3	2.70	CL

*C*_u_, coefficient of uniformity; *C*_c_, coefficient of gradation; %clay, clay content; %silt, silt content; %sand, sand content; γ_d_, unit weight; PL, plastic limit; LL, liquid limit; G_s_, specific gravity; USCS, Unified soil classification system.

**Figure 2:**
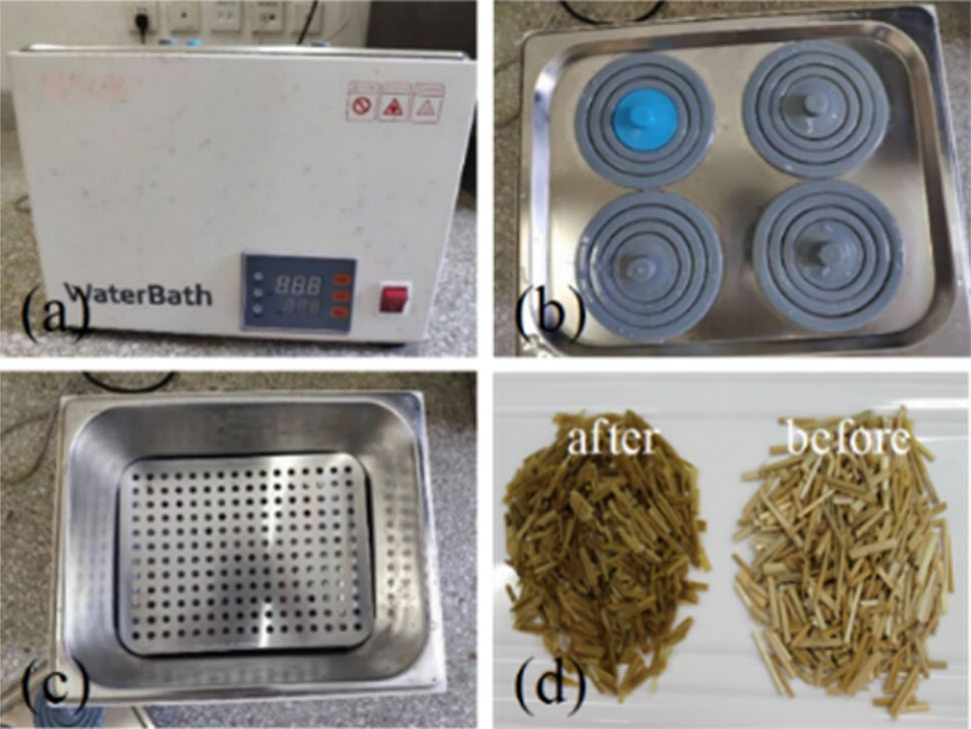
Water bath apparatus: (**a**) control panel, (**b**) sub water tanks, (**c**) heater, and (**d**) waste straw before and after the boiled water treatment.

**Figure 3:**
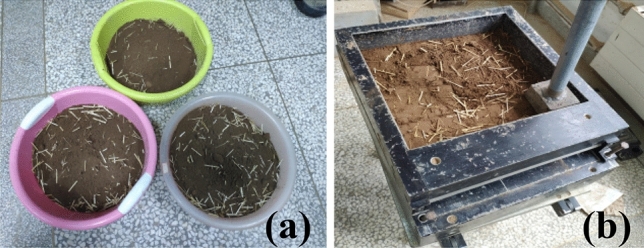
(**a**) loess-straw mixture and (**b**) reinforced specimen fabrication.

While preparing the large, reinforced specimens, straw fibers were trimmed to 40 mm long and treated with boiled water (see Fig. 2). %waste straw = 0.6 and water content *ω* = 18%, resulting from the displacement-controlled direct shear test results, were then fed back to the preparation of the specimens applied to the stress-controlled direct shear and creep tests^4,49,50^. The loess-straw fiber mixture was loaded in the shear box in three batches, and each batch was compacted using the moist tamping technique (see Fig. 3), reducing the specimens’ non-homogeneity^51^.

### Testing apparatus and methods

**Figure 4:**
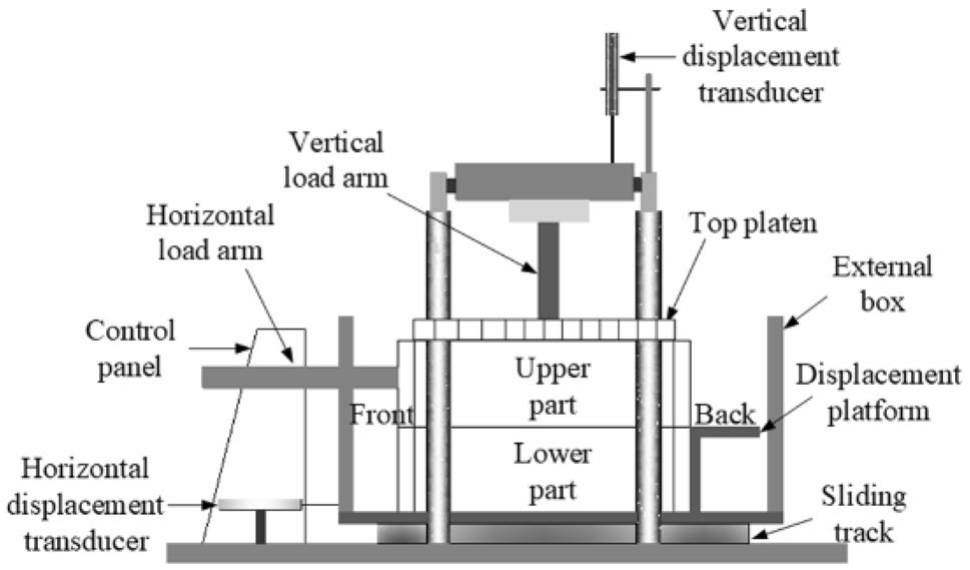
Schematic illustration of the direct shear test apparatus.

The large-scale direct shear test apparatus of Geocomp ShearTrac-III was used to determine the shearing behavior of the unreinforced and reinforced loess specimens (Fig. 4). The vertical and horizontal load and displacement applied to the unreinforced and reinforced specimens are controlled by the micro-stepper motors of Geocomp ShearTrac-III. Two additional vertical displacement transducers installed respectively at the front and back of the shear box are to measure the top platen rotation of the shear box during shearing. The unreinforced and reinforced specimens were sheared starting from a shear stress of 8.3 kPa with an increment of 8.3 kPa under the normal pressure *σ*_n_ = 50 kPa, 75 kPa, 100 kPa, respectively, until the failure state was attained where the specimen horizontal and vertical displacements were increased very sharply^52^. The failure state is defined as a change in curvature of the relation of horizontal displacement *δ*_h_ versus elapsed time t from positive to negative and a rapid increase in *δ*_h_ value. The testing program applied to the stress-controlled direct shear tests is shown in Table 2.

**Table 2:** Direct shear and creep testing programmes.

Direct shear test name	Sample name	Normal stress *σ*_n_ (kPa)	Stress ratio (*R* = *τ*/*τ*_50_)
Stress-controlled direct shear test	DS_0_, DS_0.6_	50, 75, 100	
Creep test	DC_0_, DC_0.6_	100, 300	0.95, 1.0, 1.02, 1.03, 1.05

*τ*, shear stress; *τ*_50_, shear stress associated with *δ*_h_ = 50 mm.

Creep test for the specimens was conducted following the displacement-controlled direct shear test. The specimens were sheared at a shear rate of 0.8 mm/min and a constant *σ*_n_ until the horizontal displacement *δ*_h_ attained 50 mm, and the shear stress associated with a horizontal displacement *δ*_h_ = 50 mm was defined as ‘large-displacement shear strength’ and applied throughout this study. During the creep tests, the specimens were subjected to different levels of shear stress to investigate their creep behavior and threshold shear stress before attaining a creep failure. The creep deformation was increased very rapidly once reached a creep failure, and a ‘long-term shear strength’ could be defined with the onset of the accumulation of the creep deformation. The testing program is also shown in Table 2. The creep tests were progressed and then terminated when the specimens reached a variation of the horizontal displacement being < 0.01mm in 24 hours^40^. To check and validate the testing method’s accuracy, repeatability tests are considered as of great necessity prior to the formal tests. For this reason, six repeatability tests on the specimens at %straw fiber = 0 and ω = 18% under σ_n_ = 100 kPa, 200 kPa, and 300 kPa respectively were conducted and documented in the work of Xue et al. (2021). The repeatability with respect to the stress-controlled direct shear and creep tests was validated via the other 12 repeatability tests (six for each test configuration) and can be compared to that present in the afore-said six repeatability tests. The results are not presented here due to limited space.

## Results

### Shear behaviour in stress-controlled direct shear tests

**Figure 5:**
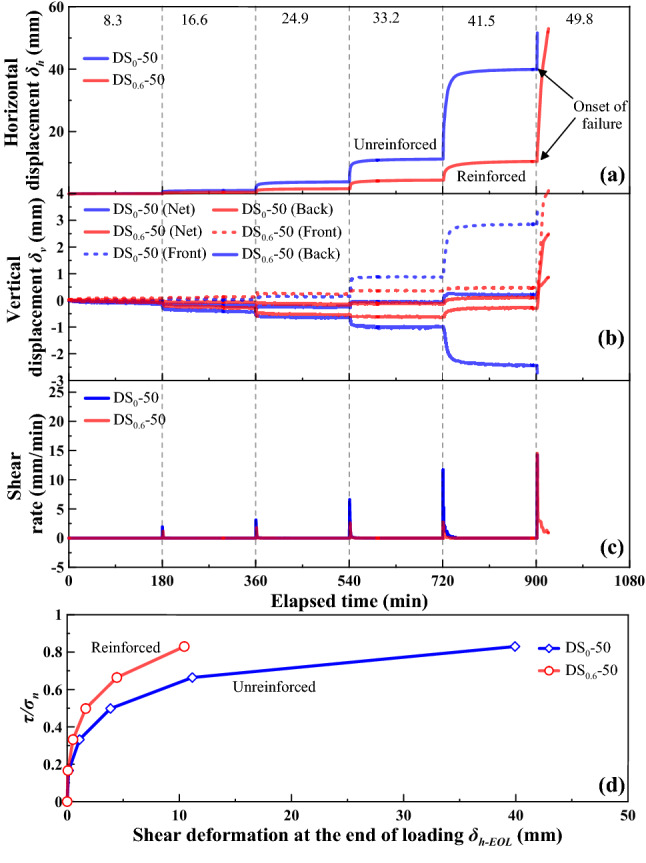
Shear behaviour for the reinforced specimen (*ω* = 18% and %waste straw = 0.6) and the unreinforced specimen (*ω* = 18%) under *σ*_n_ = 50 kPa: (**a**) shear displacement (*δ*_h_) versus elapsed time relationship, (**b**) vertical displacement (*δ*_v_) versus elapsed time relationship (**c**) shear rate versus elapsed time relationship, and (**d**) ratio of shear stress to total normal stress (*τ*/*σ*_n_) versus shear displacement at the end of each increment of shear load (*δ*_h-EOL_) relationship.

**Figure 6:**
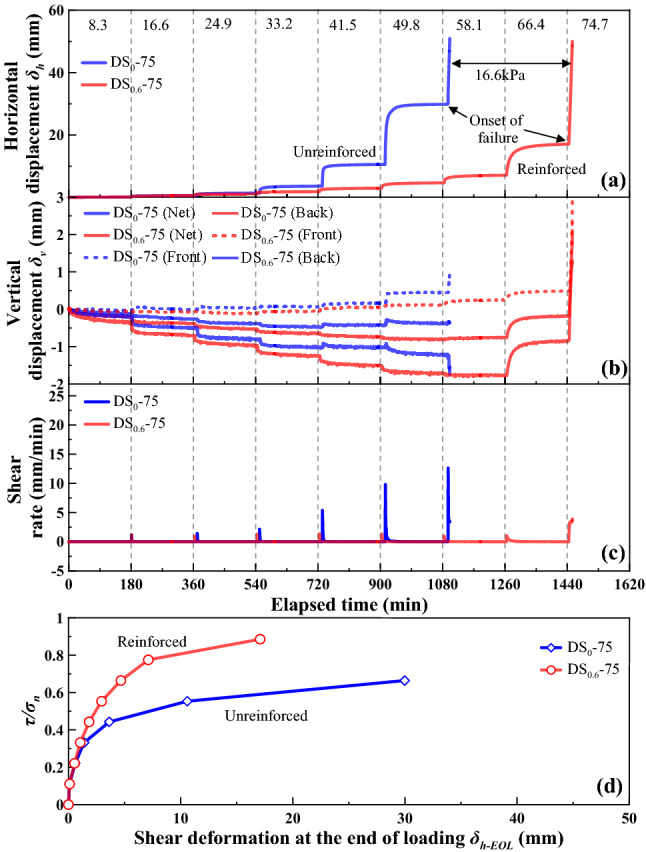
Shear behaviour for the reinforced specimen (*ω* = 18% and %waste straw = 0.6) and the unreinforced specimen (*ω* = 18%) under *σ*_n_ = 75 kPa: (**a**) shear displacement (*δ*_h_) versus elapsed time relationship, (**b**) vertical displacement (*δ*_v_) versus elapsed time relationship (**c**) shear rate versus elapsed time relationship, and (**d**) ratio of shear stress to total normal stress (*τ*/*σ*_n_) versus shear displacement at the end of each increment of shear load (*δ*_h-EOL_) relationship.

**Figure 7:**
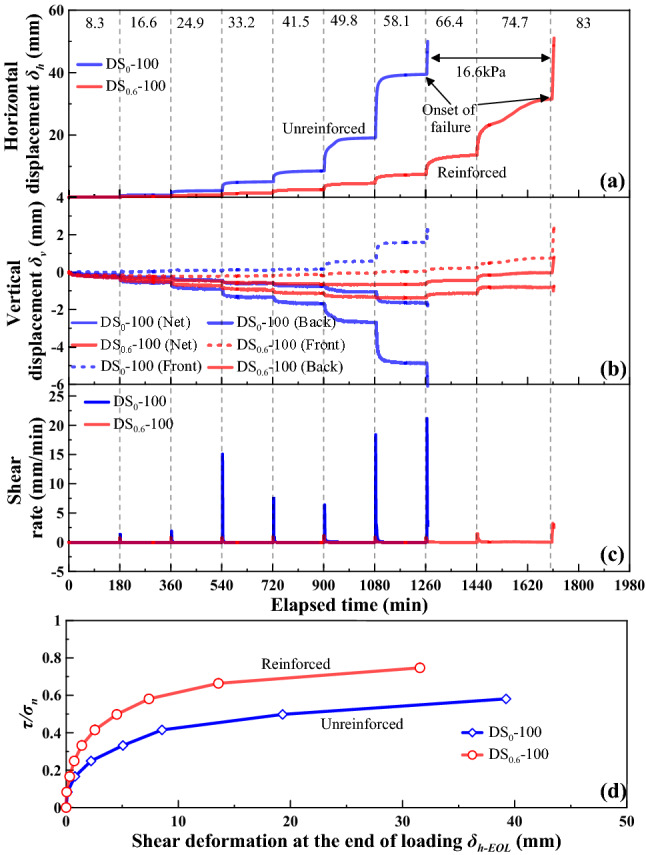
Shear behaviour for the reinforced specimen (*ω* = 18% and %waste straw = 0.6) and the unreinforced specimen (*ω* = 18%) under *σ*_n_ = 100 kPa: (**a**) shear displacement (*δ*_h_) versus elapsed time relationship, (**b**) vertical displacement (*δ*_v_) versus elapsed time relationship (**c**) shear rate versus elapsed time relationship, and (**d**) ratio of shear stress to total normal stress (*τ*/*σ*_n_) versus shear displacement at the end of each increment of shear load (*δ*_h-EOL_) relationship.

The shear behaviour for the loess-straw reinforcement mixture specimens at *ω* = 18%, with %waste straw = 0 and 0.6, under *σ*_n_ = 50 kPa, 75 kPa, and 100 kPa is shown in Figs. 5, 6 and 7, respectively, as the following four relationships: (a) horizontal displacement (δ_h_) versus elapsed time; (b) vertical displacement (*δ*_v_) versus elapsed time; (c) shear rate versus elapsed time; and (d) the ratio of shear stress to normal pressure (*τ*/*σ*_n_) versus horizontal displacement at the end of each increment of shear load (*δ*_h-EOL_). Vertical dashed lines shown in Figs. 5, 6 and 7 represent an increase in *τ* of 8.3 kPa and the value adjacent to each line represents the applied shear stress. *δ*_h_ and *δ*_v_ values generally increased with the increasing values of shear stress *τ*. Prior to a failure, there were two phases observed in the development of *δ*_h_ and *δ*_v_, namely rapid primary shear phase and stable secondary shear phase^9,26^. The development of *δ*_h_ was significant and rapid in the very beginning of each shear load stage, referred also to as the rapid primary shear phase, followed by the development of *δ*_h_ and *δ*_v_ at a nearly constant shear rate, referred also to as the stable secondary shear phase. The unreinforced specimen has accumulated larger horizontal displacements before it reaches failure in comparison with the reinforced one. The discrepancy in the horizontal displacement *δ*_h_ that was required for attaining a failure between the reinforced and unreinforced specimen was especially significant at greater normal pressures. Further, the shear rate increased notably in the very beginning of each shear load stage and then declined to approximately zero. Moreover, the *τ*/*σ*_n_ versus *δ*_h-EOL_ curve for the reinforced specimen lay above the *τ*/*σ*_n_ versus *δ*_h-EOL_ curve for the unreinforced specimen. The slope of the *τ*/*σ*_n_ versus *δ*_h-EOL_ curve progressed sharply initially and then steadily declined with the increasing *δ*_h-EOL_ until a shear failure was attained.

Since the specimen front may heave and the specimen back may settle throughout the shearing, there were three types of vertical displacement, namely *δ*_v_ (front), *δ*_v_ (back), and *δ*_v_ (net), to be measured in this study, as shown in Figs. 5, 6 and 7. The development of *δ*_v_ (front) and *δ*_v_ (back) was closely related to the top platen rotation problem^53^. *δ*_v_ (net) was, in fact, measured from the specimen center, irrespective of the top platen rotation problem. It was the smallest amongst the measurements. Further, the curve slope changed from negative to positive, especially for *δ*_v_ (back) and *δ*_v_ (net). This was also likely to be due to the top platen rotation problem and would be discussed later in this paper.

### Creep behaviour in creep tests

**Figure 8:**
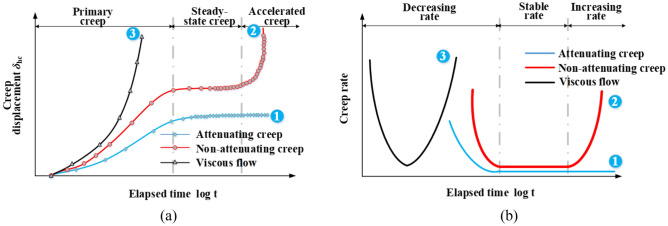
Three typical creep curves: (**a**) creep displacement versus elapsed time, (**b**) creep rate versus elapsed time.

There were three typical creep deformation-elapsed time relationships identified in this study, namely, (1) attenuating creep: the shear rate gradually decreased with the development of creep displacement and finally shifted to nearly zero (i.e. a variation of *δ*_hc_ being smaller than 0.01 mm in 24 hours) (see curve 1 in Fig. 8); (2) non-attenuating creep: it was characterised by three distinct stages: primary creep, steady-state creep, and accelerated creep. The shear rate decreased to a minimum in the primary creep stage and remained constant in the steady-state creep stage. Finally, the shear rate increased rapidly, corresponding to an accumulation of the creep displacement *δ*_hc_ (see curve 2 in Fig. 8); (3) viscous flow: the creep displacement *δ*_hc_ accumulated rapidly in a short period of time in the initial creep stage, showing a brittle failure behavior (see curve 3 in Fig. 8).

**Figure 9:**
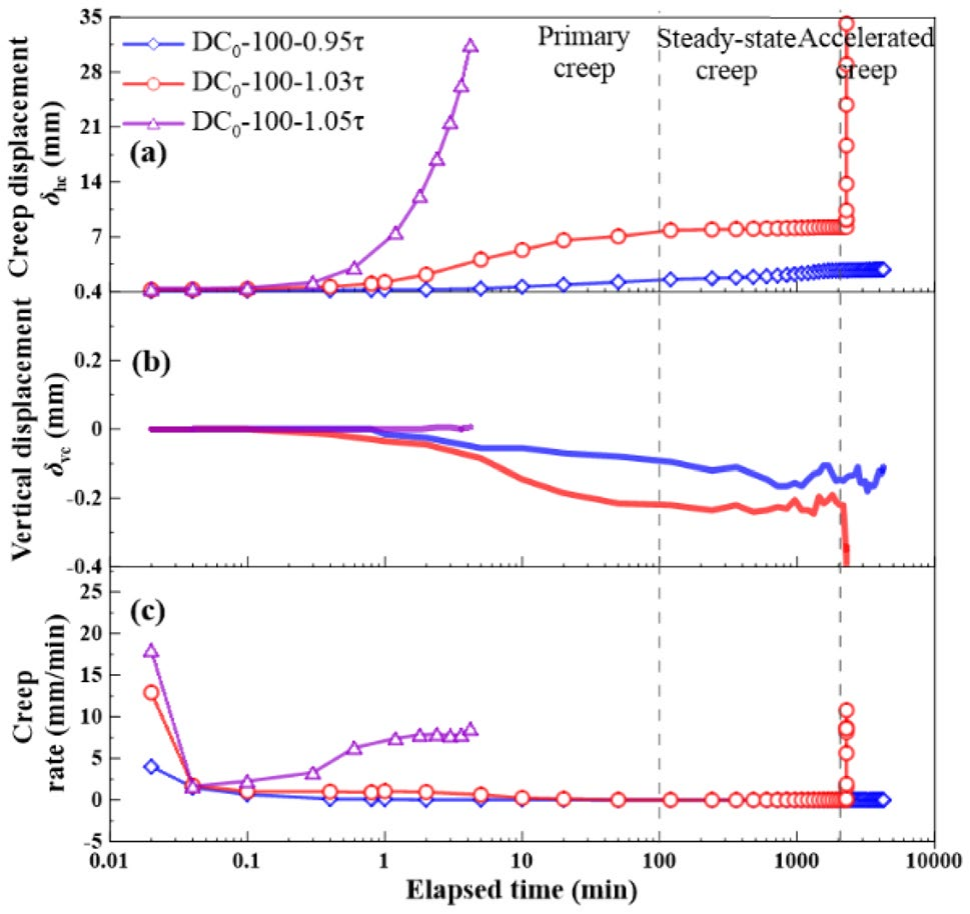
Relationships of (**a**) creep displacement (*δ*_hc_) versus elapsed time, (**b**) vertical displacement (*δ*_vc_) versus elapsed time, and (c) creep rate versus elapsed time for the unreinforced specimens at *ω* = 18% under *σ*_n_ = 100 kPa.

**Figure 10:**
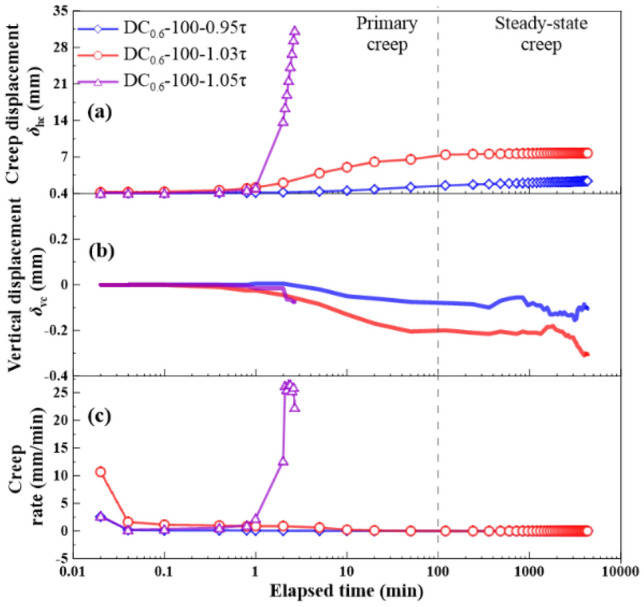
Relationships of (**a**) creep displacement (*δ*_hc_) versus elapsed time, (**b**) vertical displacement (*δ*_vc_) versus elapsed time, and (c) creep rate versus elapsed time for the reinforced specimens at *ω* = 18% under *σ*_n_ = 100 kPa.

**Figure 11:**
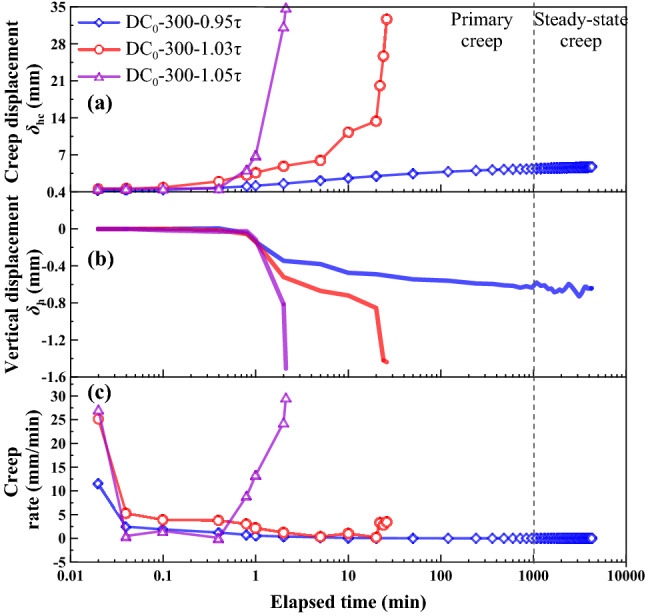
Relationships of (**a**) creep displacement (*δ*_hc_) versus elapsed time, (**b**) vertical displacement (*δ*_vc_) versus elapsed time, and (c) creep rate versus elapsed time for the unreinforced specimens at *ω* = 18% under *σ*_n_ = 300 kPa.

**Figure 12:**
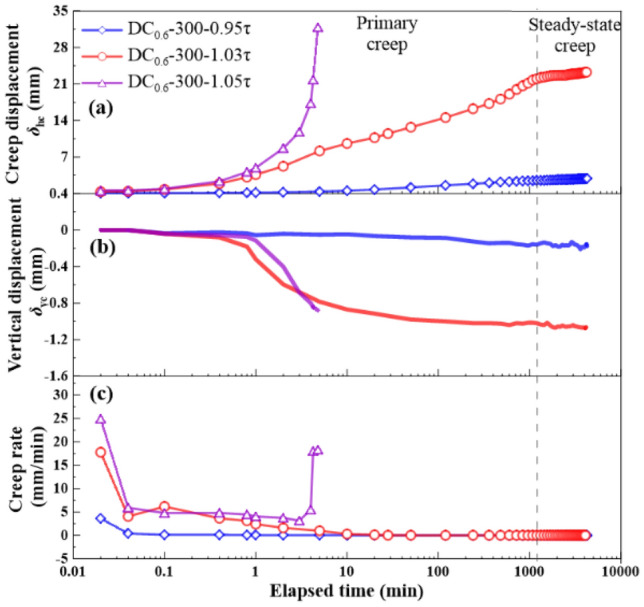
Relationships of (**a**) creep displacement (*δ*_hc_) versus elapsed time, (**b**) vertical displacement (*δ*_vc_) versus elapsed time, and (c) creep rate versus elapsed time for the reinforced specimens at *ω* = 18% under *σ*_n_ = 300 kPa.

The shear stress ratios *R* = 0.95, 1.0, 1.02, 1.03, and 1.05 were applied to the creep tests. *R* was defined as a ratio of shear stress *τ*_r_ in creep stage to large-displacement shear strength *τ*_ld_ and expressed as:

1 $$R = \tau _{{\text{r}}}{/}\tau _{{{\text{ld}}}}$$

where *R* = shear stress ratio, *τ*_r_ = shear stress in creep stage, *τ*_ld_ = large-displacement shear strength. The creep behavior is shown in Figs. 9, 10, 11 and 12, as the following three relationships: (a) *δ*_hc_ versus elapsed time; (b) *δ*_vc_ versus elapsed time; (c) shear rate versus elapsed time. Due to limited space, the results only associated to *R* = 0.95, 1.03, and 1.05 were presented in this study. All the unreinforced and reinforced specimens were featured by the steady-state creep after passing the primary creep stage when subjected to *R* = 0.95. The steady-state creep for the unreinforced and reinforced specimens commenced since the elapsed time was longer than 100 min, which also indicated the completion of the rearrangement of soil particles. Furthermore, the creep displacement *δ*_hc_ accumulated very quickly in the primary creep stage when subjected to *R* = 1.05, indicating that all of the unreinforced and reinforced specimens were featured by a viscous flow character. The time required for the unreinforced specimens to reach 30 mm was shorter than the reinforced specimens; the unreinforced specimens required only 2 min to reach *δ*_hc_ = 30 mm and the reinforced specimens require 5 min to reach *δ*_hc_ = 30 mm. Moreover, the unreinforced specimen under *σ*_n_ = 100 kPa was featured by non-attenuating creep when subjected to *R* = 1.03, whereas the reinforced specimen was, however, featured by attenuating creep. In contrast to *σ*_n_ = 100 kPa, the unreinforced specimen under *σ*_n_ = 300 kPa showed a viscous flow character, whereas the reinforced specimen presented an attenuating creep character. The straw reinforcement appeared to provide the ability to resist the accumulation of *δ*_hc_ in the accelerated creep stage for the reinforced specimens, promoting the formation of the attenuating creep character. The lack of the straw reinforcement for the unreinforced specimens caused some difficulty in resisting the accumulation of *δ*_hc_ either in the primary creep stage or in the accelerated creep stage.

## Discussion

### Shear behaviour in stress-controlled direct shear tests

The temporal relationships of *δ*_h_ showed the effect of straw reinforcement on the shear behavior of the reinforced specimen during the stress-controlled direct shear tests (Figs. 5, 6, 7). The value of *δ*_h_ for a given shear load was greater in the unreinforced specimen compared to the reinforced specimen. This difference was attributed to a combination of the distribution of shear stresses along the straw length and the tensile elongation of the straw reinforcement. A rapid accumulation of *δ*_h_ and a change in curve slope from positive to negative indicated a failure state of specimen. The unreinforced and reinforced specimen under *σ*_n_ = 50 kPa reached their failure state when subjected to a shear load of 49.8 kPa. The unreinforced specimen under *σ*_n_ = 75 kPa reached its failure state when subjected to a shear load of 58.1 kPa, whereas under the same normal pressure, the reinforced specimen reached its failure when subjected to a shear load of 74.7 kPa. The shear load upon failure of specimen was increased with the increasing *σ*_n_, and the difference in the shear load upon failure between the unreinforced specimen and the reinforced specimen appeared to be enlarged when subjected to higher *σ*_n_. The rapid accumulation of *δ*_h_ indicated that the shear resistance reached a maximum, which corresponded to the shear strength. In other words, the rapid accumulation of *δ*_h_ was accompanied by the onset of shear failure. In spite that the initiation of shear failure triggered the distribution of shear stresses amongst the remaining reinforcement straws, *τ* could not be resisted as the shear resistance started losing after the initiation of shear failure.

The positive value of *δ*_v_ (net), measured as the average of *δ*_v_ (front) and *δ*_v_ (back), indicated expansion during shearing, whereas the negative value indicated compression. The development of *δ*_v_ (net) (i.e., expansion) for the unreinforced specimen was more significant under *σ*_n_ = 50 kPa and *σ*_n_ = 75 kPa compared to the reinforced specimen (Figs. 5b and 6b). However, the development of *δ*_v_ (net) for the reinforced specimen was more significant under *σ*_n_ = 100 kPa compared to the unreinforced specimen (Fig. 7b). It could be considered that expansion of the reinforced specimen was suppressed by lower *σ*_n_. Under higher *σ*_n_, particles had limited space to proceed relative movement during shearing. On the other hand, the development of *δ*_v_ (front) and *δ*_v_ (back) was aggravated not only by the increasing *σ*_n_ but also by the top platen rotation. The development of *δ*_v_ (net) and *δ*_v_ (back) was somehow suppressed by the inversed top platen rotation when the curve slope changed from negative to positive (see Figs. 5, 6, 7).

The *τ*/*σ*_n_ versus *δ*_h-EOL_ relationships in Figs. 5, 6 and 7 highlighted the difference in shear behavior between the unreinforced specimen and the reinforced specimen. The *τ*/*σ*_n_ versus *δ*_h-EOL_ curve for the reinforced specimen lay above the *τ*/*σ*_n_ versus *δ*_h-EOL_ curve for the unreinforced specimen. The effect of straw reinforcement caused smaller *δ*_h-EOL_ prior to failure. In contrast, the lack of straw reinforcement caused larger *δ*_h-EOL_ prior to failure. Furthermore, the reinforced specimen could withstand higher τ than the unreinforced specimen for the same horizontal displacement.

### Effect of shear stress ratio on creep behavior

**Figure 13:**
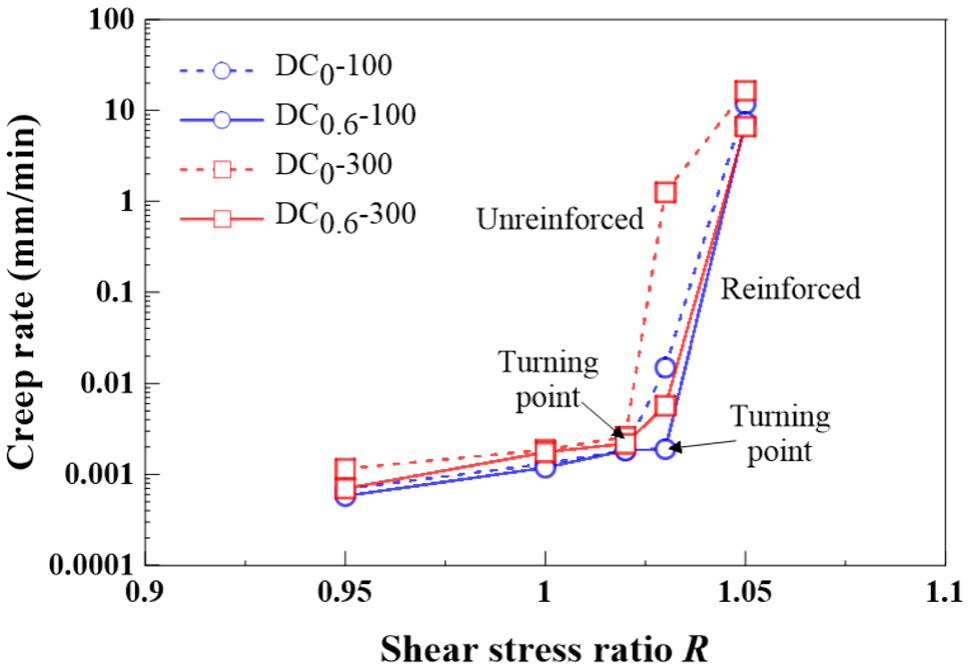
Creep rate-shear stress ratio relationships for the unreinforced and reinforced specimens under *σ*_n_ = 100 kPa and *σ*_n_ = 300 kPa.

The creep displacement *δ*_hc_ versus elapsed time relationships in Figs. 9, 10, 11 and 12 show different creep behaviors including attenuating creep, non-attenuating creep, and viscous flow. However, it was still a long way from a better understanding of the mechanism of triggering the creep behaviors^54,55^. Hence, an important factor, the shear stress ratio, may help to improve our understanding of the formation mechanism of the creep behaviors^44^. It is worth noting that a single data point present in Fig. 13 corresponds to a ratio of maximum creep displacement to elapsed time, resulting from a curve present in Figs. 9, 10, 11 and 12 with the shear stress ratio same as that data point has. Figure 13 shows the creep rate versus shear stress ratio R relationships for the unreinforced and reinforced specimens under *σ*_n_ = 100 kPa and *σ*_n_ = 300 kPa. The creep rate versus shear stress ratio *R* relationships for the unreinforced and reinforced specimens were featured by a bilinear character. The first section ranging from *R* = 0.95 to *R* = 1.02 was formed for the unreinforced specimens under *σ*_n_ = 100 kPa and *σ*_n_ = 300 kPa, followed by the second section ranging from *R* = 1.02 to *R* = 1.05. In contrast to the unreinforced specimens, the first section for the reinforced specimens formed in a range of *R* = 0.95 to *R* = 1.03, followed by the second section in a range of *R* = 1.03 to *R* = 1.05. Similarly, the curve for the reinforced specimen under *σ*_n_ = 100 kPa lay above the curve for the reinforced specimen under *σ*_n_ = 300 kPa. These results indicated that the reinforced specimen was subjected to the shear stress ratio *R* higher than the unreinforced specimen prior to the rapid accumulation of creep rate (i.e., specimen failure). Furthermore, the creep rate for the reinforced specimens was smaller than the unreinforced specimens under the same *R*. The shear stress in creep stages caused the interparticle contacts to loosen, leading to particle rearrangement. The effect of straw reinforcement was highlighted by imposing some difficulty in progressing particle relative movement during particle rearrangement. In addition to the difficulty, the straw fibers as soil reinforcement elements notably contributed to the increase in shear resistance by distributing shear stresses exerted in the shear plane along their length. The enhanced shear resistance, induced by the effect of straw reinforcement, also supported the fact that the reinforced specimen under *σ*_n_ = 100 kPa and *σ*_n_ = 300 kPa behaved in an attenuating creep character when subjected to *R* = 1.03 compared to the unreinforced specimen behaving in a non-attenuating creep character or viscous flow when subjected to the same *R* (Figs. 9, 10, 11, 12).

### Determination of long-term shear strength

In addition to the shear stress ratio, long-term shear strength was also deemed crucial in determining the creep behaviour. The long-term shear strength is the minimum strength under which the interparticle contacts start losing progressively, which can lead to particle rearrangement and ultimately result in failure. Macroscopically, the long-term shear strength is defined as the ultimate strength observed in a creep test and corresponds to the maximum shear stress^56^. It can be used as a criterion to judge the creep failure of specimens.

**Figure 14:**
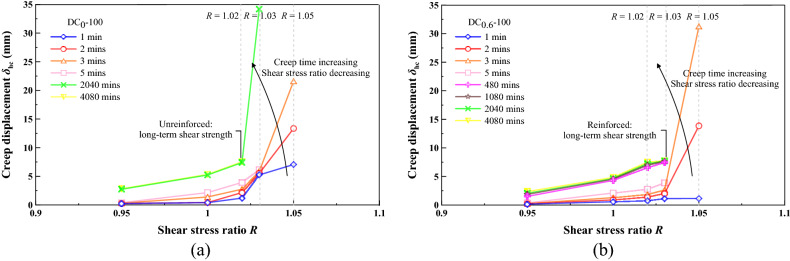
Isochronal curve for (**a**) unreinforced specimen under *σ*_n_ = 100 kPa and (**b**) reinforced specimen under *σ*_n_ = 100 kPa.

**Figure 15:**
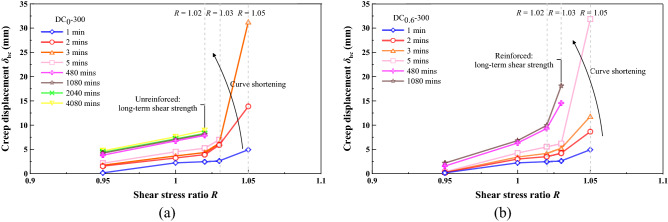
Isochronal curve for (**a**) unreinforced specimen under *σ*_n_ = 300 kPa and (**b**) reinforced specimen under *σ*_n_ = 300 kPa.

The long-term shear strength is the threshold value that distinguishes the attenuating creep from the non-attenuating creep and could be determined by the turning point of isochronal curve, as recommended by Wang^57^. The isochronal curve may be capable of deepening our understanding of the long-term shear strength. Figures 14 and 15 shows the isochronal curves for the unreinforced and reinforced specimens under *σ*_n_ = 100 kPa and *σ*_n_ = 300 kPa. The isochronal curves of the unreinforced and reinforced specimens look differently, and they are all nonlinear, which also indicates the nonlinear rheological properties. The isochronal curves were nearly overlap at low *R*, indicating that the time effect on the creep behaviour could be neglected at low *R* values (Fig. 14). In contrast to low *R* values, the isochronal curves were not identical at high *R* values, and their difference was enlarged with the increasing *R* values. Further, the longer the creep time, the smaller the *R* value; for the unreinforced specimen under *σ*_n_ = 100 kPa, the isochronal curve upon 1 min after the onset of the creep test terminated at *R* = 1.05, whereas the isochronal curve upon 4080 mins ended up with *R* = 1.02. This also indicated a time-dependent deterioration of the shear strength within the shear plane, induced by the interparticle contact loss resulting from the shear stresses in the creep test. According to the characters of the isochronal curves, the long-term shear strength can be characterised as an isochronal curve possessing the longest creep time and the smallest *R* value. Given that *R* is defined as a ratio of *τ*_r_ to *τ*_ld_, it can simply be introduced to backcalculate the long-term strength. For the unreinforced specimen under *σ*_n_ = 100 kPa, it corresponded to the isochronal curve upon 4080 mins after the commencement of the creep test. The long-term shear strength for the unreinforced specimen can, therefore, be backcalculated using *R* = 1.02 (i.e. curve turning point). In other words, the long-term shear strength for the unreinforced specimen under *σ*_n_ = 100 kPa was 1.02 times the large-displacement shear strength. The shortest isochronal curve for the reinforced specimen under the same normal pressure was identical to the isochronal curve upon 5 mins after the beginning of the creep test. The long-term shear strength can be backcalculated using *R* = 1.03. Further, the isochronal curves upon a creep time longer than 5 mins were not shortened any further, indicating that the long-term shear strengths remained nearly constant, most likely because of the enhanced shear strength, induced by the effect of straw reinforcement. Figure 15 shows the isochronal curves for the unreinforced and reinforced specimens under *σ*_n_ = 300 kPa. The isochronal curve upon 480 mins after the onset of the creep test terminated at *R* = 1.02 for the unreinforced specimen, which also indicated the long-term shear strength. For the reinforced specimen, the isochronal curve upon 480 mins after the beginning of the creep test ended up with *R* = 1.03. *R* = 1.03 can be used for determining the long-term shear strength for the reinforced specimen towards achieving a prediction of slow-moving landslides with a pre-existing shear surface. These results also indicate that countermeasure is in pressing need when the sliding mass initiates its downwards movement. The longer the time taken for executing the countermeasure, the more likely the potential of having shear stresses in excess of the long-term strength (i.e. creep failure), and the more likely the landslide hazard will occur.

### Comparison of long-term shear strength and peak shear strength

**Figure 16:**
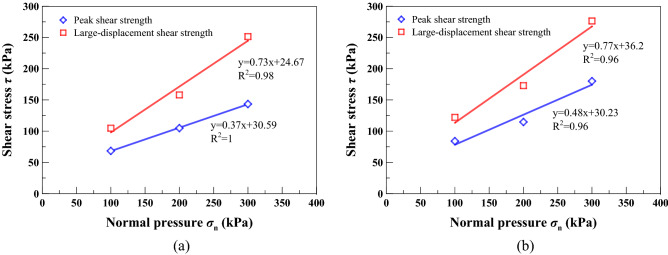
Shear strength envelopes: (**a**) unreinforced specimens and (**b**) reinforced specimens.

The long-term shear strength parameters for the unreinforced and reinforced specimens were determined through the failure envelopes shown in Fig. 16 and compared to the peak and large-displacement shear strength parameters towards highlighting the creep behaviour. It can be seen from Table 3 that the shear strengths are generally ordered as: long-term shear strength > large-displacement shear strength > peak shear strength. The data of peak strength present in Fig. 16 are, in fact, derived from the displacement-controlled direct shear tests done by Xue et al. (2021). The stress-strain relationships for the reinforced specimens generally behaved in a strain-hardening manner, and therefore, this made the large displacement strength (corresponding to the shear strain being 16.7%) higher than the peak strength (derived when the shear strain reaches approximately 6.5%). Further, the reinforced specimens possessed a superior performance in limiting the creep displacement than the unreinforced specimens. The creep behaviour represents a time-dependent mechanical character^58^ and can, therefore, be better interpreted using the isochronal curves. Although the creep behaviour varied notably in creep stages, the creep test provided a period of time, allowing the soil to regain and even further elevate the shear strength by dissipating the excess porewater pressure in the shear plane. In addition to the time effect, the mutual inlay in the shear plane and the interparticle bonding also elevated the shear strength within the shear plane. On the other hand, the effect of straw reinforcement caused the large-displacement, long-term, and peak shear strength parameters of the reinforced specimen greater than those of the unreinforced specimen. Some difficulty in progressing particle relative movement, induced by the presence of straw reinforcement, and shear stress distribution along the length of straw fibers were deemed to be the main contributor leading to the enhanced shear strength for the reinforced specimen. On the whole, the creep test results highlighted the importance of the long-term shear strength and explored the exciting potential for the use of straw reinforcement for guiding the design of high-filled embankment slopes. Further, the use of long-term shear strength was confirmed effective in managing the risk of embankment slope instability at the lowest possible cost^59^.

**Table 3:** Summary of the peak, large-displacement, and long-term shear strength parameters.

Specimen	Shear box (mm)	Type of shear strength	%waste straw (%)	*c′* (kPa)	*ϕ′* (°)
Quaternary loess	305	Peak shear Strength	0	30.59	20.30
			0.60	24.67	36.13
		Large-displacement Shear strength	0	36.45	36.50
			0.60	30.23	25.64
		Long-term shear strength	0	36.20	37.50
			0.60	47.75	38.00

Huangtupo landslide is a typical reactivated slow sliding landslide^42^. Its progressive sliding can be compared to the non-attenuating creep character for the unreinforced specimen subjected to *R* = 1.03 and *σ*_n_ = 100 kPa. The sliding mass initially slided at a higher displacement rate in a primary stage and subsequently a lower displacement rate in a creep stage. In a creep test, the shear stresses given caused a chain reaction of interparticle contact loss, leading to the accumulation of interparticle relative movement within the shear band^60^. Such chain reaction was accompanied with excessive viscous displacement, rapid weakening of creep resistance, and eventually accelerated creep displacement. These ultimately led to a new slow-moving landslide^61^ and were considered to be the main cause leading to the Huangtupo landslide^42^.

## Conclusions

In this study, the unreinforced and reinforced loess specimens were sheared under the stress-controlled direct shear test and the creep test. Three creep behaviours including attenuating creep, non-attenuating creep, and viscous flow were identified. The long-term shear strength, induced by the creep test, was compared to the peak shear strength. Based on the results and discussion, some main conclusions can be drawn as follows:

1. The temporal relationships of *δ*_h_ highlighted the effect of straw reinforcement on the shear behavior of the reinforced specimen (see Figs. 5, 6, 7). The difference in *δ*_h_ between the unreinforced specimen and the reinforced specimen was attributed to a combination of the distribution of shear stresses along the straw length and the tensile elongation of the straw reinforcement. The greater *δ*_h_ for the unreinforced specimen than the reinforced specimen provided testimony of the afore-said statement. Aa a result, the large-displacement, long-term, and peak shear strength parameters of the reinforced specimen were greater than those of the unreinforced one. Some difficulty in progressing particle relative movement, induced by the presence of straw reinforcement, and shear stress distribution along the length of straw fibers, was considered as the main contributor to the enhanced shear strength for the reinforced specimen. Furthermore, the difference in the failure load between the unreinforced and reinforced specimen appeared to be enlarged with the increase of *σ*_n_. Moreover, τ could not be resisted any further because the shear strength starts losing after the initiation of failure. On the other hand, the development of *δ*_v_ (front) and *δ*_v_ (back) was aggravated not only by the increasing *σ*_n_ but also by the top platen rotation.
2. There were three creep characters identified in this study, including the attenuating creep, non-attenuating creep, and viscous flow. *δ*_hc_ versus elapsed time relationships indicated that the unreinforced and reinforced specimen behaved in a different manner when subjected to the same shear stress ratio *R*. The reinforced specimen was subjected to the shear stress ratio *R* higher than the unreinforced specimen prior to the rapid accumulation of *δ*_hc_. The creep rate versus shear stress ratio *R* relationships confirmed the observation.
3. Isochronal curve can be used not only for presenting the time-dependent deterioration of the shear strength within the shear plane but also for determining the long-term shear strength. The long-term shear strength can be characterised as an isochronal curve possessing the longest creep time and the smallest shear stress ratio *R*. The time effect appeared to have a minimal effect on the creep behaviour at a low *R* range. Further, the reinforced specimen was featured by R greater than the unreinforced specimen under the same *σ*_n_. The chain reaction of interparticle contact loss, accompanied with excessive viscous displacement, rapid weakening of creep resistance, and eventually accelerated creep displacement, could be the main cause leading to a new slow-moving landslide (e.g., Huangtupo landslide).

## Data Availability

The experimental data used to support the findings of this research work are included in the article.

## List of symbols

*C*_u_: Coefficient of uniformity
*C*_c_: Coefficient of gradation
*G*_s_: Specific gravity
γ_d_: Dry unit weight
PL: Plastic limit
LL: Liquid limit
*ω*: Water content
*σ*_n_: Normal stress
*R*: Shear stress ratio
*δ*_h_: Horizontal displacement
*δ*_hc_: Creep displacement
*δ*_v_: Vertical displacement
*δ*_vc_: Vertical displacement in creep stage
*δ*_h__-EOL_: Horizontal displacement at the end of each increment of shear load
*τ*: Shear stress
*τ*_50_: Shear stress associated with *δ*_h_ = 50 mm
*τ*_r_: Shear stress in creep stage
*τ*_ld_: Large-displacement shear strength
